# Cost-utility analysis of add-on vericiguat for the treatment of chronic heart failure with reduced ejection fraction in China

**DOI:** 10.1186/s12889-024-18778-2

**Published:** 2024-05-09

**Authors:** Lu Wang, Xuechen Huo, Haiyan Sun, Feiyu Liu, Ruiqin Huang, Quan Zhao

**Affiliations:** 1 Department of Pharmacy, Yantai Yuhuangding Hospital, Yantai, Shandong Province 264000 China; 2 Department of Hepatological Surgery, Yantai Yuhuangding Hospital, Yantai, Shandong Province 264000 China

**Keywords:** Markov model, Vericiguat, VICTORIA clinical trial, Cost-utility, Heart failure

## Abstract

**Objective:**

This study aimed to evaluate the cost-utility of the addition of vericiguat for treating chronic heart failure (CHF) in China from the healthcare payer’s perspective.

**Methods:**

A Markov model was built to estimate the cost and utility of treating CHF using vericiguat plus standard treatment (vericiguat group) vs. standard treatment alone (standard treatment group). The clinical parameters (mortality of cardiovascular and hospitalization rate of HF) were calculated according to the VICTORIA clinical trial. The HF cost and utility data were obtained from the literature published in China. One-way sensitivity analysis and probability sensitivity analysis were performed.

**Results:**

According to the 13-year model, vericiguat was more expensive (155599.07 *CNY* vs. 259396.83 *CNY*) and more effective (4.41 *QALYs* vs. 4.54 *QALYs*). The incremental cost‐utility ratio (ICUR) was 802389.27 *CNY* per *QALY*. One-way sensitivity analysis revealed that cardiovascular mortality in the two groups was the parameter that had the greatest impact on the results. The GDP per capita in 2022 in China was 85,700 *CNY*. The probability sensitivity analysis (PSA) showed that the probability of vericiguat being cost-effective was only 41.7% at the willingness-to-pay (WTP) threshold of 3 times GDP per capita (257,100 *CNY*).

**Conclusions:**

In China, the treatment of CHF with vericiguat is not cost-effective. The drug price could decrease to 145.8 *CNY*, which could be considered cost-effective.

**Supplementary Information:**

The online version contains supplementary material available at 10.1186/s12889-024-18778-2.

## Introduction

Heart failure (HF) is the severe and terminal stage of various heart diseases [1]. HF remains a major health problem worldwide, with a substantial economic burden [2]. The global prevalence of HF in adults is 1 − 3%, and the average incidence in China is 1.1% [3]. The incidence of HF gradually increases with age. The prevalence of HF is 0.57% for the population aged between 25 and 64 years in China. However, the incidence increases to 3.86% among individuals aged 65 to 74 years and to 7.55% among individuals aged over 80 years. The overall cost of HF is estimated to reach $108 billion per year and is expected to increase as the economy develops and the global population grows [4, 5]. In China, the average cost of HF for outpatients was $892.3, which was much greater than the mean outpatient fee ($551.6) in 2019 [3, 6]. Strengthening the prevention and management of HF and reducing the financial burden are crucially important [6, 7].

Vericiguat was approved for treating HF by the U.S. Food and Drug Administration (FDA) in January 2021 and by the National Medical Products Administration (NMPA) in China on May 18, 2022. Vericiguat is the first soluble guanylate cyclase (sGC) stimulator used for the treatment of HF; it can directly stimulate sGC independently from NO and has a synergistic effect on NO [8]. The results of the phase 3 clinical trial (VICTORIA clinical trial) showed that the incidence of the primary end point (death due to cardiovascular causes or first hospitalization for HF) was decreased in the vericiguat group compared to that in the standard treatment group. There was no significant difference in the incidence of adverse events between the vericiguat group and the standard treatment group in the VICTORIA clinical trial [9].

In addition to effectiveness and safety, economy is equally important. However, there have been reports on the cost-effectiveness analysis and budget impact analysis of vericiguat in the United States [10, 11]. Given the economic burden of HF, it is necessary to carry out a pharmacoeconomic evaluation of vericiguat based on the Chinese national situation. This study aimed to estimate the cost-utility of adding vericiguat to standard treatment in China’s medical setting from the perspective of Chinese health care payers.

## Methods

Cost-effectiveness analysis (CEA) and cost-utility analysis (CUA) are the most common methods used for pharmacoeconomic evaluation [12]. CUA outcomes are expressed by the total cost, quality‐adjusted life year (QALYs), and the incremental cost‐utility ratio (ICUR (cost per *QALY*)) [12, 13]. A Markov model was constructed for the CUA in this study to estimate the cost-utility of adding vericiguat to standard treatment for HF.

### Target population and overview

The VICTORIA trial was used as the basis for the data in the Markov model in this CUA. The target population has been previously reported in detail in the VICTORIA trial [9]. A total of 5050 HF patients with New York Heart Association (NYHA) classification II to IV and left ventricular ejection fraction (LVEF) < 45% in 42 countries were recruited for the VICTORIA trial. The median follow-up was 10.8 months.

The CUA was conducted from the perspective of the Chinese health care payer. This study analyzed two therapeutic schedules: vericiguat plus standard treatment (vericiguat group) and standard treatment alone (standard treatment group). Patients in the vericiguat plus standard treatment (vericiguat group) were also treated with vericiguat at 2.5 mg/d for the first 2 weeks, followed by 5 mg/d for the third and fourth weeks and 10 mg/d thereafter. The Markov model was constructed to be 13 years with 3-month cycles, based on the mean age at baseline of 67.3 years of the VICTORIA clinical trial and the maximum average life expectancy of 80.88 years in China [14]. The costs are valued at 2022 CNY. Cost and utility were all discounted at 5.0% annually for 13 years. Only direct costs were considered in this study. The outcomes were the total cost, QALYs, and ICUR.

### Markov model

**Fig. 1:**
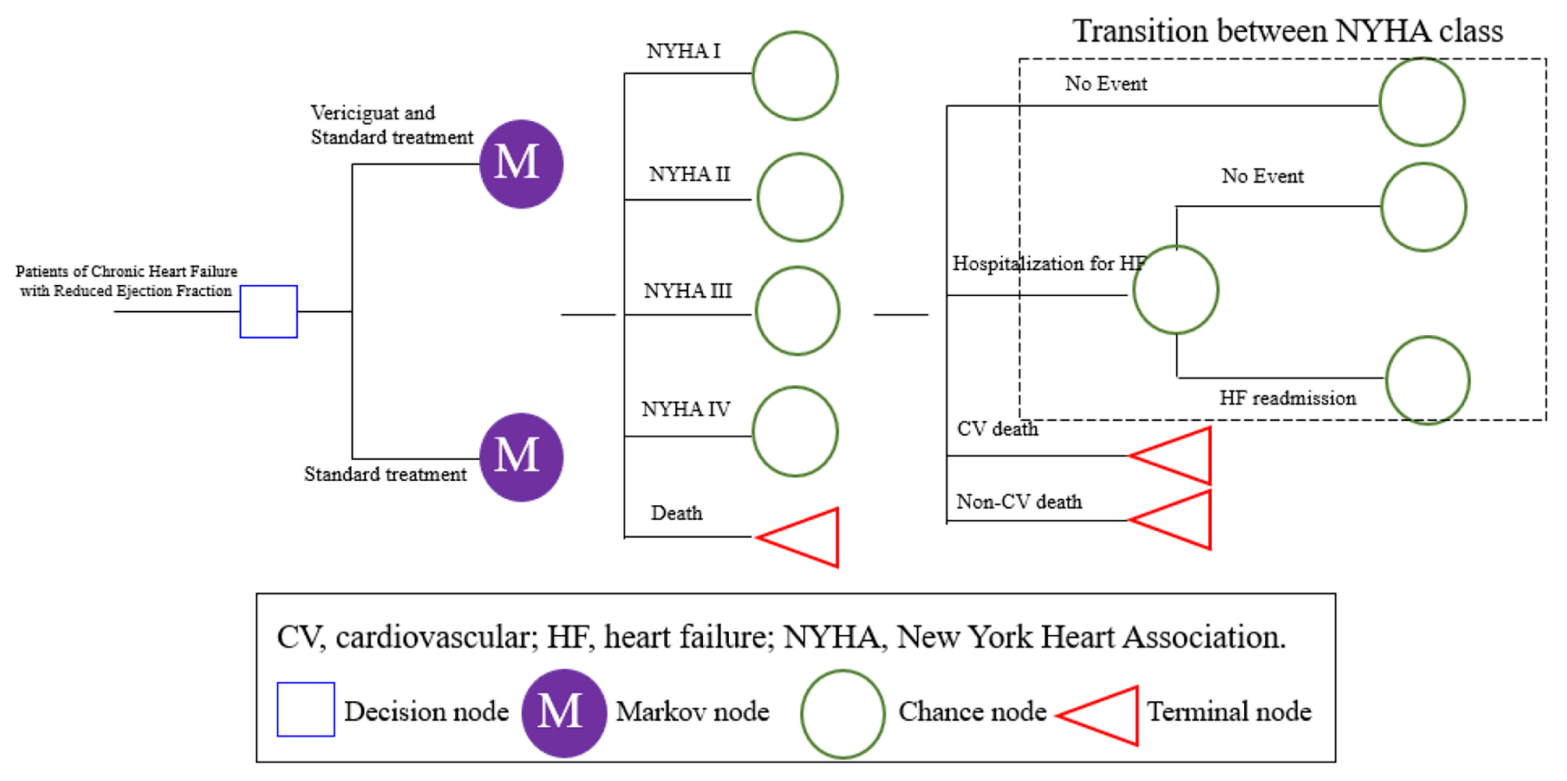
Markov model structure

The Markov model was used to perform a CUA (Fig. 1). All patients could have five disease states (NYHA I-IV and death). In each cycle, simulated patients could transition between different NYHA classes or experience death. Patients with NYHA I-IV could experience four events—no event, hospitalization for HF, cardiovascular (CV) death, and non-CV death—in each cycle. In addition, we assumed that all patients who experienced HF hospitalization could experience HF readmission within 3 months because of the increased risk of readmission in the vulnerable period [15]. Patients were transferred to the next cycle at different NYHA classes. A half-cycle correction was implemented in the model to prevent the overestimation of expected survival. Model building and analyses were performed with TreeAge Pro 2022.

### Input parameters

#### Clinical parameters

The initial distribution of the NYHA class was based on the VICTORIA clinical trial (I: 0.0%, II: 59.0%, III: 39.7%, and IV: 1.3%) [9]. The 3-month transition probabilities between different NYHA classes are shown in Table 1 [16]. The CV mortality and HF hospitalization event rates were calculated according to the VICTORIA clinical trial, which was converted to 3‐month probabilities (Table 2) [9, 16]. Non-CV mortality was deduced by the Chinese Center for Disease Control and Prevention (CDC) [17].

**Table 1:** Transition probabilities among NYHA class (3 months)

To	NYHA I	NYHA II	NYHA III	NYHA IV	Distribution
From					
NYHA I	0.977	0.019	0.004	0.000	Dirichlet
NYHA II	0.008	0.981	0.010	0.001	Dirichlet
NYHA III	0.000	0.034	0.960	0.006	Dirichlet
NYHA IV	0.000	0.000	0.055	0.945	Dirichlet

**Table 2:** The occurrence rate in the model of 3 months

Parameters (3 months)		Base Value	Range	Distribution	Data Sources
Event rate					
Vericiguat group					
Mortality of CV		2.33%	2.21 − 2.45%	Beta; α = 2.33; β = 97.67	VICTORIA Clinical Trial [9]
HF hospitalization		8.49%	8.07 − 8.91%	Beta; α = 8.49; β = 91.51	VICTORIA Clinical Trial [9]
HF readmission		76.99%	None	None	VICTORIA Clinical Trial [9]
Standard treatment group					
Mortality of CV		2.56%	2.43 − 2.69%	Beta; α = 2.56; β = 97.44	VICTORIA Clinical Trial [9]
HF hospitalization		9.29%	8.83 − 9.75%	Beta; α = 9.29; β = 90.71	VICTORIA Clinical Trial [9]
HF readmission		78.85%	None	None	VICTORIA Clinical Trial [9]
Mortality of non-CV of two groups	68–70	0.244%	None	None	Chinese CDC [17]
	71–75	0.312%	None	None	Chinese CDC [17]
	76–80	0.450%	None	None	Chinese CDC [17]

HF: heart failure; CV: cardiovascular

#### Cost parameters

The costs in this study included only drug acquisition costs and hospitalization costs. Indirect costs were not investigated in this study. The costs of hospitalization for HF were derived from a real-world study in 2017 in China [18]. The cost of basic HF medications, including ACEIs, ARBs, *β*‐blockers, and MRAs, was 906 *CNY* for 3 months, as calculated based on literature published in 2017 [19]. The costs were converted based on the inflation values of 104.3%, 102.4%, 101.8%, 100.4%, and 100.6% into 2022 [14]. The prices of vericiguat were 406 *CNY* (14 tablets per box, 5 mg per tablet) and 238.84 *CNY* (14 tablets per box, 2.5 mg per tablet) in 2022 [20]. The dosage of vericiguat was 2.5 mg for 2 weeks, and the dose was increased to 5 mg and ultimately to the target dose of 10 mg once daily [9]. The costs of vericiguat were 3892.84 *CNY* for the first three months and 4872 *CNY* every three months thereafter. The costs of the vericiguat group included the costs of vericiguat and basic medications (Table 3).

**Table 3:** Cost and utility data used in the model (3 months)

Parameters (3 months)		Base Value	Range	Distribution	Data Sources
Cost					
Standard treatment (*CNY*)		994.93	895.44–1094.42	Gamma; Mean = 994.93; SD = 50.76	Fu et al. [19]
Vericiguat and Standard treatment (*CNY*)	the first three months	4887.77	5280.24-6453.62	Gamma; Mean = 5866.93; SD = 299.33	Bidding price [20]
	the other three months	5866.93			Bidding price [20]
Hospitalization for HF (*CNY*)	NYHA I	7624.03	6861.63–8386.43	Gamma; Mean = 7624.03; SD = 388.98	Xuan et al. [14]
	NYHA II	28657.14	25791.43–31522.85	Gamma; Mean = 28657.14; SD = 1462.10	Xuan et al. [14]
	NYHA III	53589.71	48230.74–58948.68	Gamma; Mean = 53589.71; SD = 2734.17	Xuan et al. [14]
	NYHA IV	46659.46	41993.52–51325.41	Gamma; Mean = 42488.95; SD = 2380.58	Xuan et al. [14]
Discounted rate		5%	0–8%	None	
Utility					
NYHA I		0.2138	0.2113–0.2160	Beta; α = 21.38; β = 78.62	Fu et al. [19]
NYHA II		0.1865	0.1803–0.1930	Beta; α = 18.65; β = 81.35	Fu et al. [19]
NYHA III		0.1485	0.1363–0.1610	Beta; α = 14.85; β = 85.15	Fu et al. [19]
NYHA IV		0.1408	0.1288–0.1525	Beta; α = 14.08; β = 85.92	Fu et al. [19]
Disutility for hospitalization					
NYHA I		-0.01	None	None	Fu et al. [19]
NYHA II		-0.02	None	None	Fu et al. [19]
NYHA III		-0.03	None	None	Fu et al. [19]
NYHA IV		-0.07	None	None	Fu et al. [19]

#### Utility parameters

The utility of 1 indicated full health, and the utility of death was 0. The utility of NYHA I, NYHA II, NYHA III, and NYHA IV was derived from research in China and deduced to 3-month values, as shown in Table 3 [19]. Considering that hospitalization can reduce quality of life, utility decreases if patients experience hospitalization, which means disutility. (Table 3).

Cost and utility were discounted at 5.0% annually for 13 years.

### Outcomes

The mean total cost and QALYs were outputted using the Markov model. The QALYs included both quantity and quality of life. Specifically, QALYs are the product of the life years in each health state and the health utility value of that state. The ICUR was the ratio of the difference in the mean cost divided by the difference in the mean QALYs. The willingness-to-pay (WTP), which refers to the maximum amount that a patient is willing to pay for a medicine or service, was used to assess cost-utility. In China, 3 GDP per *QALY* is a commonly accepted WTP threshold [21]. When one treatment was more expensive and more useful than the other treatment, the ICUR and WTP were applied to measure whether the expensive treatment showed cost-utility. The expensive treatment could be the cost-utility strategy if the ICUR was less than the WTP.

### Sensitivity analysis

One-way sensitivity analysis and probability sensitivity analysis (PSA) were performed to examine how the model parameters affected the results. The parameter variation ranges used in the one-way sensitivity analysis are shown in Tables 2 and 3. The cost of standard treatment, vericiguat, and HF hospitalization ranged from 10% above to 10% below the baseline value. The discount rate ranged from 0 to 8%. CV mortality and the rate of HF hospitalization increased and decreased by 5%, respectively. The feasible range of utility of NYHA I, NYHA II, NYHA III, and NYHA IV was set according to the literature (Table 3**)** [19]. The parameters associated with the PSA were CV mortality and incidence of HF hospitalization in the two groups; utility of NYHA I, NYHA II, NYHA III, and NYHA IV; and the cost of standard treatment, vericiguat, and HF hospitalization. The Monte Carlo simulation of the PSA was conducted for 1000 iterations.

## Results

### Model validation and base case

The CV mortality and hospitalization rates when the model was operated for 9 months and 12 months are shown in Figure S1. The results indicated that the calculated values of our model agreed well with the results of the VICTORIA clinical trial [22].

The results of the costs, QALYs, and the ICUR of the cost-utility analysis are shown in Table 4. In the 13-year model, vericiguat was more effective but was also more expensive. The total costs of the 13‐year-circulation were 155599.07 *CNY* and 259396.83 *CNY* in the standard treatment group and the vericiguat group, respectively. The vericiguat group was associated with 4.54 *QALY*s, which was 0.13 *QALY*s greater than that of the standard treatment group. The GDP per capita in 2022 in China was 85,700 *CNY* [14]. Compared with that of the standard treatment group, the ICUR of the vericiguat group was 802389.27 *CNY* per *QALY*, which exceeded the WTP of 257,100 *CNY* per *QALY* (3 times GDP per capita) in China in 2022.

**Table 4:** The results of cost-utility analysis of the vericiguat plus standard treatment vs. standard treatment

Treatment	Total costs (CNY)	Quality-adjusted life year (QALYs)	Mean life year	Incremental cost (CNY)	Incremental QALY	ICUR (CNY per QALY)
vericiguat plus standard treatment	259396.83	4.54	6.65	103797.76	0.13	802389.27
standard treatment	155599.07	4.41	6.48			\

### One way sensitivity analysis

**Fig. 2:**
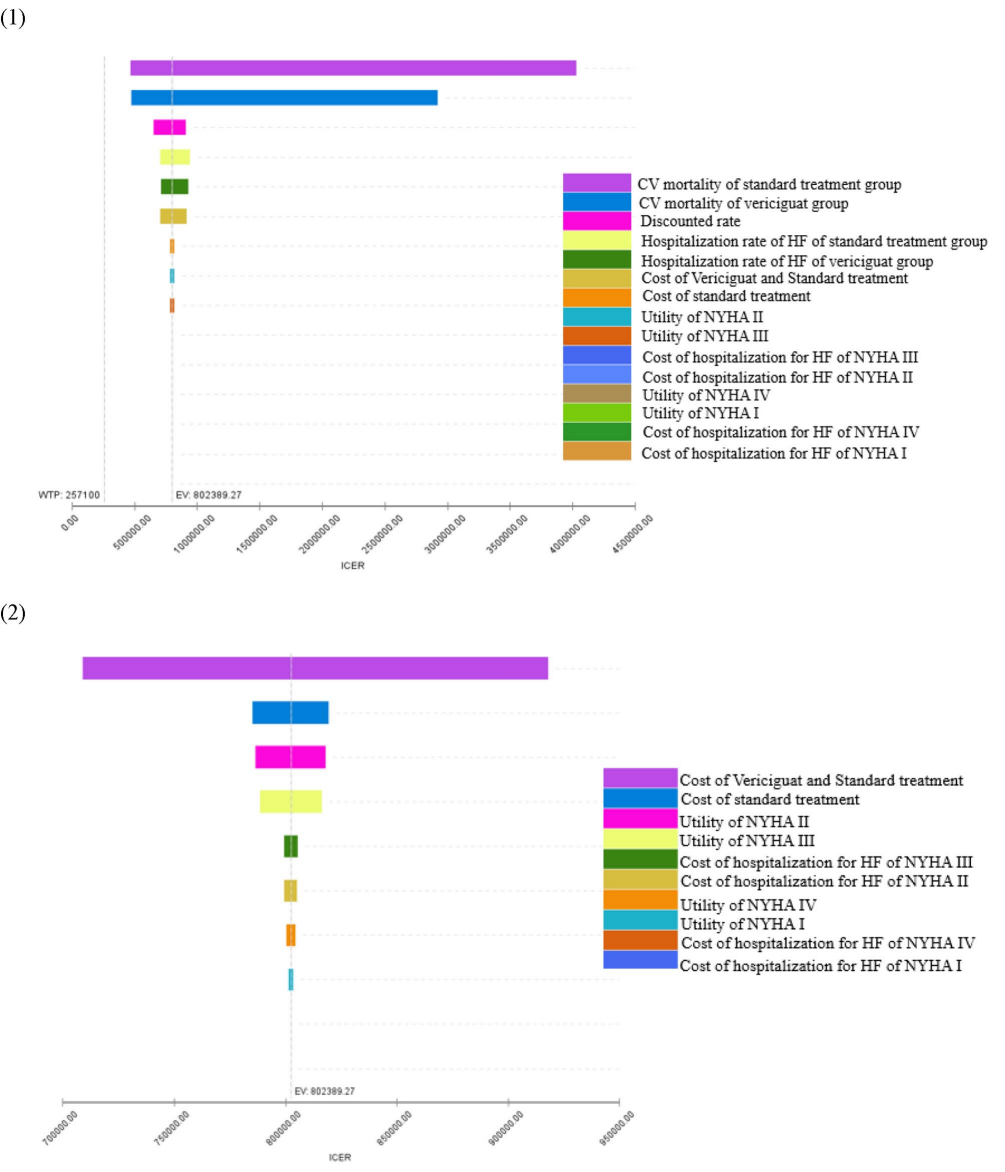
Tornado graphs of one-way sensitivity analysis

The results of the one-way sensitivity analysis are shown in Table 5; Fig. 2. The results indicated that CV mortality in the two groups was the parameter that had the greatest impact on the results. However, even at the lower limit of CV mortality in the vericiguat group and the upper limit of CV mortality in the standard treatment group, the corresponding ICUR was 478713.94 *CNY* per *QALY* and 466863.30 *CNY* per *QALY*, respectively, which was still greater than the WTP of 257,100 *CNY* per *QALY*. The one-way sensitivity analysis indicated that the results were robust. (Table 5)

**Table 5:** The results of one-way sensitivity analysis of the vericiguat plus standard treatment vs. standard treatment

Input Variable		Range	ICUR (CNY per QALY)
Cost of Standard treatment (*CNY*)		895.44–1094.42	785379.05–819399.48
Cost of Vericiguat and Standard treatment		5280.24-6453.62	708990.42–918106.09
Cost of Hospitalization for HF (*CNY*)	NYHA I	6861.63–8386.43	802344.3–802441.02
	NYHA II	25791.43–31522.85	799484.27–805294.26
	NYHA III	48230.74–58948.68	799359.58–805418.95
	NYHA IV	41993.52–51325.41	802385.8–802761.67
Discounted rate		0–8.0%	652792.32–914072.51
Utility	NYHA I	0.2113–0.2160	801397.01–803519.81
	NYHA II	0.1803–0.1930	786549.8–818103.78
	NYHA III	0.1363–0.1610	788493.38–816432.21
	NYHA IV	0.1288–0.1525	800422.14–804416.9
Rate of HF hospitalization for vericiguat group		8.07 − 8.91%	713894.66–932928.01
CV mortality for vericiguat group		2.21 − 2.45%	478713.94–2924302.69
Rate of HF hospitalization for standard treatment group		8.83 − 9.75%	709621.5–942558.34
CV mortality for standard treatment group		2.43 − 2.69%	466863.30–4030027.00

### Probability sensitivity analysis

**Fig. 3:**
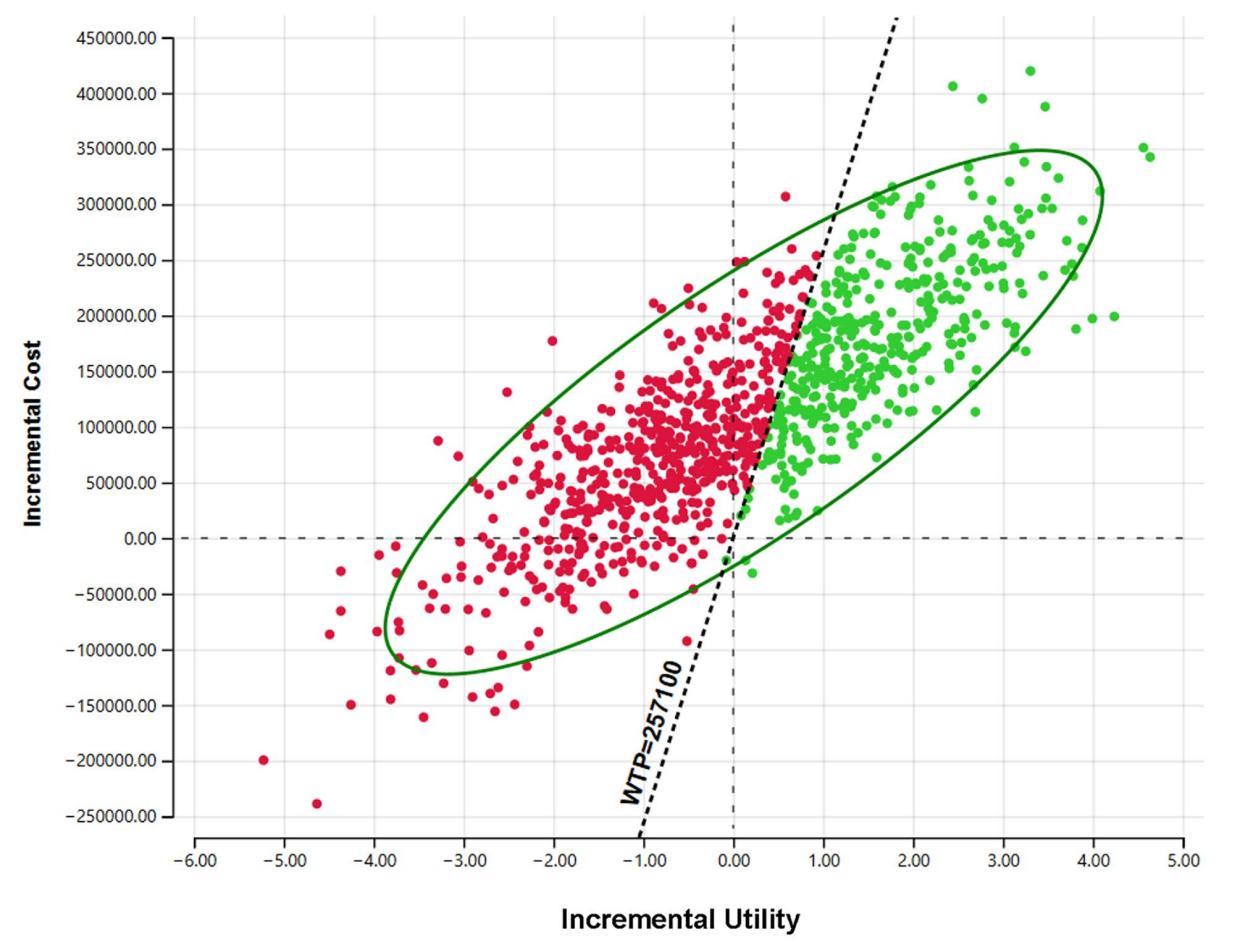
The incremental cost-utility (ICUR) scatter plot

**Fig. 4:**
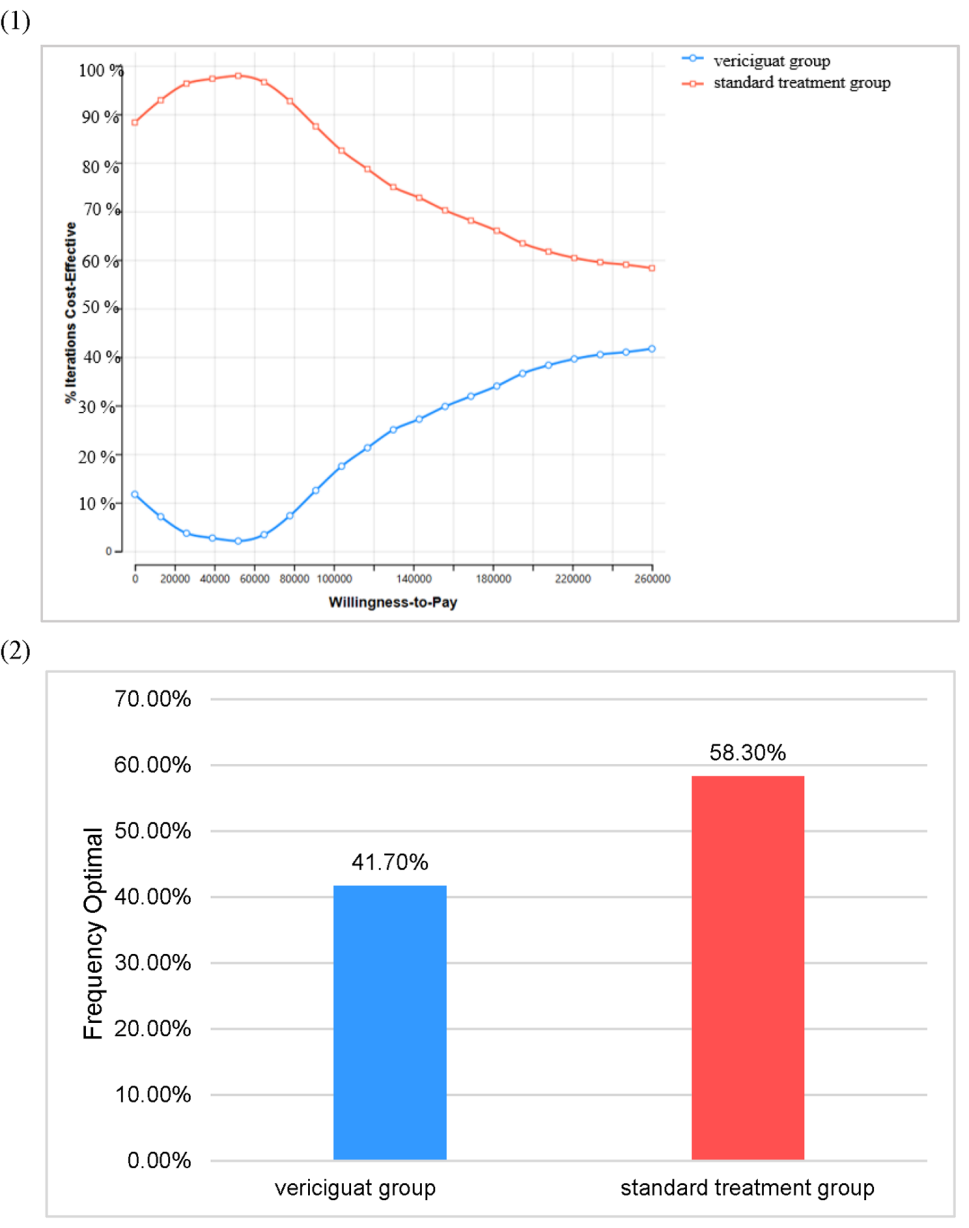
(1) C-E acceptability curve. (2) Economic probability of two groups at a WTP threshold of 257,100 *CNY*.

The ICUR scatter plot was obtained by 1000 Monte Carlo Simulation (Fig. 3). The horizontal coordinate is the incremental utility, and the vertical coordinate is the incremental cost. The upper-right corner of the axis indicates that vericiguat treatment was more expensive and more effective. The top left corner shows that vericiguat was more expensive and less effective. The diagonal line across the image is the incremental cost-utility ratio curve at 257,100 *CNY*. When the scatter point is on the right side of this line, vericiguat may be economical for patients. The C-E acceptability curve (Fig. 4) derived from the PSA showed that at a WTP threshold of 257,100 *CNY* per *QALY*, vericiguat plus standard treatment had only 41.7% probability of being considered to have cost-utility.

## Discussion

To the best of our knowledge, there has been no pharmacoeconomic evaluation of vericiguat for HF treatment in China [10, 11]. A Markov model was constructed to simulate the progression of CHF and to evaluate the cost and health outcomes associated with two different therapeutic approaches. The study population of VICTORIA clinical trial consisted of individuals with HF with reduced ejection fraction (HFrEF) who had experienced a recent HF hospitalization for less than 6 months or who received outpatient intravenous diuretic therapy for less than 3 months [9]. The findings of this investigation revealed that vericiguat was both more costly and more effective in the treatment of the overall intent-to-treat population of the VICTORIA clinical trial over a 13-year simulation. By incorporating data from the VICTORIA clinical trial data into the model, the results demonstrated that during the 13 years of cyclic simulations, the addition of vericiguat to standard treatment resulted in an additional 0.13 *QALYs* with 802389.27 *CNY* per *QALY*. This cost was significantly greater than the WTP threshold of 257,100 *CNY* per *QALY*. These findings suggested that vericiguat was not a cost‐utility treatment option.

The robustness of this conclusion was confirmed through both PSA and one-way sensitivity analyses. The results of the one-way sensitivity analysis clearly and consistently indicated that adding vericiguat was not cost-utility. In the one-way sensitivity analysis, in which the parameters of CV mortality of the two groups, the HF hospitalization rate for the two groups, the utility, the cost of standard treatment, the cost of vericiguat, and the cost of hospitalization for HF were changed, the vericiguat group was consistently not a cost-utility therapeutic method, as the ICUR much greater than the WTP of 257,100 *CNY* per *QALY*. The results also showed that the CV mortality of the two groups had the strongest impact on the outcome. In the VICTORIA clinical trial, the hazard ratio (HR) of primary outcome events in the Asian subgroup was slightly larger than that in the total cohort (HR 0.91 (0.75–1.11) and 0.90 (0.82–0.98)). Therefore, the difference in CV mortality between the two groups of Asian patients in the VICTORIA trial may be less than that in our study, which indicated that the ICUR in Asian patients seems to be larger than that shown in our analysis.

The PSA results indicated that at a WTP threshold of 257,100 *CNY*, there was only a 41.7% likelihood that the vericiguat group would be considered to be cost-utility, further supporting the notion that vericiguat is not cost-utility at present prices.

The results of both PSA and one-way sensitivity analyses indicated that adding vericiguat to standard treatment did not show cost-utility even if the price was reduced by 10%. Therefore, a substantial reduction in the price of vericiguat is imperative to achieve cost-utility. The estimation of the price of vericiguat was based on a WTP value of 257,100 *CNY* per *QALY* gained, which is 3 times the per capita GDP of China in 2022. Additionally, a value of 85,700 *CNY* per *QALY* (the per capita GDP in 2022) was used to calculate the price of vericiguat [23]. A price of 145.8 *CNY* per box containing 5 mg of vericiguat in 14 tablets was considered to cost-utility at a WTP of 257,100 *CNY* per *QALY*. Furthermore, if the ICUR decreased to 85,700 *CNY* per *QALY*, the drug price could decrease to 65.7 *CNY* and still be considered cost-utility.

The 1–3 GDP per capita per QALY was chosen as the threshold ICUR of the countries that do not have a specific threshold ICUR. In this study, the 3 GDP per capita per QALY was used as the threshold ICUR. Due to the differences in the level of economic development in various regions of China, there are significant differences in per capita GDP among different regions. Decision-making departments in different regions can choose the appropriate scheme according to the data of this study. Patients with different payment capabilities can also make different choices according to the data of this study.

This study suggested that vericiguat was not a cost-utility treatment option from the perspective of China’s health care payers. This is important for health care policy because economics is one of the keys to entering the “Basic Medical Insurance Drug Catalogue” [24]. HF is a chronic disease for which patients incur huge treatment costs. For ordinary people, the daily cost of medications may impose a large economic burden. Therefore, it is important for patients with modest financial conditions to carefully identify the possible benefits of treatment with the addition of the vericiguat. When using vericiguat, clinicians should carefully consider its safety, effectiveness, and economy. When prescribing medication, it is important to consider both the price and effectiveness of the drug to find a cost-effectiveness, suitable, and safe drug or therapeutic schedule for patients. This also conforms to and promotes the reform of the National Medical Insurance Payment System in China, which plays an important role in saving clinical drug resources.

This study investigated the cost of vericiguat from the perspective of cost-utility, which provides some reference for the price. Drug prices should be set so that the interests of insurance institutions, pharmaceutical companies, patients, and the whole society can be reasonably distributed. The results of the pharmacoeconomic evaluation were used to establish a reasonable range of prices and to guide the management of drug prices. Future real-world studies of vericiguat in the Chinese population are needed. Policy-makers should invite experts from various aspects of pharmaceutical production, drug distribution, social security, and supervision and administration departments and make use of the research results of pharmacoeconomics to set reasonable prices for vericiguat [25, 26].

However, this study has several limitations. First, our work was performed based on the Chinese circumstances. The parameters of cost and utility may vary from city to city, and the results should be interpreted cautiously. Second, CV mortality and the rate of HF hospitalization in our research were deduced according to the VICTORIA clinical trial. The event rate was not derived from the Chinese population, and further external validation of the current model with real-world Chinese data is necessary. Third, there was no significant difference in the incidence rate of adverse drug reactions (ADRs) (serious adverse events occurred in 32.8% of the patients in the vericiguat group and 34.8% of the patients in the placebo group) in the VICTORIA clinical trial, and the occurrence of ADRs was not considered in the model. Finally, the results of this model were based on the VICTORIA trial. The Markov models may not reflect reality; for example, compliance with medication was not considered in the analysis. More clinical trials in populations with more extensive heterogeneity are needed.

## Conclusion

Our findings indicated that the addition of vericiguat in the treatment of HF may not be cost-utility in China. The drug price could decrease to 145.8 *CNY* (WTP threshold of 257,100 *CNY* per *QALY*) or 65.7 *CNY* (WTP threshold of 85,700 *CNY* per *QALY*) per box (5 mg per tablet, 14 tablets per box) and can be considered cost-utility. This study’s findings may provide recommendations for physicians and other health care professionals on the resource allocation of vericiguat. More real-world and Chinese population-based data are needed to perform more CUAs associated with vericiguat.

### Electronic supplementary material

Below is the link to the electronic supplementary material.

Supplementary Material 1

## Data Availability

This is a secondary study conducted using available evidence and all data and evidence obtained are included in article. The datasets used during the current study available from the corresponding author (Quan Zhao) on reasonable request.

## Abbreviations

CHF: Chronic heart failure
HF: Heart failure
FDA: U.S. Food and Drug Administration
NMPA: National Medical Products Administration
sGC: Soluble guanylate cyclase
CUA: Cost-utility analysis
CEA: Cost-effectiveness analysis
LYG: Life-Years-Gained
ICUR: Incremental cost-utility ratio
QALY: Quality-adjusted life year
WTP: Willingness-to-pay
PSA: Probability sensitivity analysis
HR: Hazard ratio
NYHA: New York Heart Association
LVEF: left ventricular ejection fraction
CV: cardiovascular
CDC: Chinese Center for Disease Control and Prevention
HFrEF: HF with reduced ejection fraction
ADR: Adverse drug reaction

## References

[1] McMurray JJV, Adamopoulos S, Anker SD, Auricchio A, Bohm M, Dickstein K (2013). ESC guidelines for the diagnosis and treatment of acute and chronic heart failure 2012. Revista Portuguesa De Cardiologia (English Edition).

[2] Tomasoni D, Adamo M, Anker MS, von Haehling S, Coats AJS, Metra M (2020). Heart failure in the last year: progress and perspective. ESC Heart Fail.

[3] Wang H, Chai K, Du M, Wang S, Cai JP, Li Y (2021). Prevalence and incidence of heart failure among urban patients in China: A National Population-based analysis. Circ Heart Fail.

[4] Cook C, Cole G, Asaria P, Jabbour R, Francis DP (2014). The annual global economic burden of heart failure. Int J Cardiol.

[5] Lesyuk W, Kriza C, Kolominsky-Rabas P (2018). Cost-of-illness studies in heart failure: a systematic review 2004–2016. BMC Cardiovasc Disord.

[6] National Healthcare Security Administration. Statistical Bulletin of the Development of National Medical Security in 2019. 2019. http://www.nhsa.gov.cn/art/2020/6/24/art_7_3268.html. Accessed 4 August 2023.

[7] Ziaeian B, Fonarow GC (2016). Epidemiology and aetiology of heart failure. Nat Rev Cardiol.

[8] Markham A, Duggan S, Vericiguat (2021). First Approval Drugs.

[9] Armstrong PW, Pieske B, Anstrom KJ, Ezekowitz J, Hernandez AF, Butler J (2020). Vericiguat in patients with heart failure and reduced ejection fraction. N Engl J Med.

[10] Alsumali A, Djatche LM, Briggs A, Liu R, Diakite I, Patel D (2021). Cost effectiveness of Vericiguat for the treatment of Chronic Heart failure with reduced ejection Fraction following a worsening heart failure event from a US Medicare Perspective. PharmacoEconomics.

[11] Alsumali A, Lautsch D, Liu R, Patel D, Nanji S, Djatche LM (2021). Budget Impact Analysis of Vericiguat for the treatment of Chronic Heart failure with reduced ejection Fraction following a worsening event. Adv Ther.

[12] Nerich V, Saing S, Gamper EM, Kemmler G, Daval F, Pivot X (2016). Cost-utility analyses of drug therapies in breast cancer: a systematic review. Breast Cancer Res Treat.

[13] Amirsadri M, Hassani A (2015). Cost-effectiveness and cost-utility analysis of OTC use of simvastatin 10 mg for the primary prevention of myocardial infarction in Iranian men. Daru.

[14] National Bureau of Statistics of China. National data. 2023. https://data.stats.gov.cn/easyquery.htm?cn=C01. Accessed 4 August 2023.

[15] Greene SJ, Fonarow GC, Vaduganathan M, Khan SS, Butler J, Gheorghiade M (2015). The vulnerable phase after hospitalization for heart failure. Nat Rev Cardiol.

[16] Yao Y, Zhang R, An T, Zhao X, Zhang J (2020). Cost-effectiveness of adding dapagliflozin to standard treatment for heart failure with reduced ejection fraction patients in China. ESC Heart Fail.

[17] National Center for Chronic and Noncommunicable Disease Control and Prevention. Chinese Center for Disease Control and Prevention. China mortality surveillance dataset 2017. Beijing:China Science and Technology; 2018. pp. 17–82.

[18] Xuan J, Zhu S, Wang S (2017). Real World Retrospective Chart Review study of the hospitalization costs and Associated Risk factors for heart failure patients in China. China Health Insurance.

[19] Fu J, Wu B, Lin H (2017). Cost-utility analysis of Ivabradine in the treatment of Chronic Heart failure in China. China Pharm.

[20] yaozhdata. Bidding price. 2023. https://db.yaozh.com/yaopinzhongbiao?comprehensivesearchcontent=vericiguat&. Accessed 13 July 2023.

[21] Edejer TT-T, Baltussen R, Adam T, Hutubessy R, Acharya A, Evans DB. Murray CJL. WHO guide to cost‐effectiveness analysis. 2010. https://www.who.int/choice/book/en/. Accessed 4 August 2023.

[22] Tang Y, Sang H. Cost-utility analysis of add-on dapagliflozin in heart failure with preserved or mildly reduced ejection fraction. ESC Heart Fail. 202;10(4):2524–33. 10.1002/ehf2.14426 PMC10375078 37290665

[23] Dranitsaris G, Truter I, Lubbe MS, Cottrell W, Spirovski B, Edwards J (2012). The application of pharmacoeconomic modelling to estimate a value-based price for new cancer drugs. J Eval Clin Pract.

[24] Liu C, liu G, Pharmacoeconomics (2019). Evaluation and application. China J Pharm Econ.

[25] Department of Health and Social Care. Pharmaceutical priceregulation scheme 2014, https://www.gov.uk/government/publications/pharmaceutical-price-regulation-scheme-2014. Accessed 17 March 2024.

[26] Hill-McManus D, Marshall S, Liu J (2021). Linked pharmacometric-pharmacoeconomic modeling and simulation in clinical drug development. Clin Pharmacol Ther.

