# Supplementary material for: Cost-utility analysis of add-on vericiguat for the treatment of chronic heart failure with reduced ejection fraction in China
Source: BMC Public Health. 2024 May 9;24:1275. doi: 10.1186/s12889-024-18778-2 (PMC11084139; doi:10.1186/s12889-024-18778-2)
Supplement: Supplementary file 1 — Supplementary Material 1 [file 12889_2024_18778_MOESM1_ESM.docx]

Table S1. Results between the Markov model and the VICTORIA clinical trial

| Outcomes (%) | Markov model (9 months) | | Markov model (12 months) | | VICTORIA clinical trial (10.8 months) | |
| --- | --- | --- | --- | --- | --- | --- |
|  | Vericiguat | Standard treatment | Vericiguat | Standard treatment | Vericiguat | Standard treatment |
| HF hospitalization | 24.8 | 27.0 | 32.7 | 35.6 | 27.4 | 29.6 |
| CV mortality | 6.7 | 7.3 | 8.8 | 9.6 | 8.2 | 8.9 |

HF = heart failure; CV = cardiovascular.
